# A practitioner-informed framework for yoga-based emotional regulation among rural adolescents

**DOI:** 10.3389/fpubh.2026.1842012

**Published:** 2026-05-18

**Authors:** M. P. Akshay Krishna, Preetha Menon

**Affiliations:** 1 Amrita School for Sustainable Futures, Amritapuri, Amrita Vishwa Vidyapeetham, Kollam, Kerala, India; 2 Amrita School of Spiritual and Cultural Studies, Amrita Vishwa Vidyapeetham, Amritapuri, Kollam, Kerala, India

**Keywords:** adolescent mental health, aggression regulation, community-based prevention, qualitative research, rural health, yoga intervention

## Abstract

**Background:**

Adolescent aggression is a significant public mental health concern associated with long-term behavioral, educational, and social outcomes. In rural and resource-constrained settings, limited access to specialized psychosocial services highlights the need for feasible, community-based preventive approaches. Yoga has been associated with improvements in emotional regulation and self-control; however, structured practitioner-informed guidance for delivering yoga in adolescent group settings, particularly in contexts involving aggression risk, remains limited.

**Objectives:**

This study aimed to (i) explore the experiential perspectives of yoga trainers involved in adolescent instruction and (ii) translate these perspectives into a practitioner-informed framework and practice-oriented guidance for delivering yoga sessions that support emotional regulation and aggression management among rural youth.

**Methods:**

A qualitative descriptive, survey-based design was employed. An open-ended, semi-structured online questionnaire was administered to 36 purposively selected yoga trainers (YT) in India with experience teaching adolescents; 31 complete responses were analyzed. Data were analyzed using hybrid deductive–inductive reflexive thematic analysis to identify themes related to yoga delivery, adolescent engagement, and emotional regulation.

**Results:**

Nine major thematic domains emerged, addressing developmental characteristics of adolescence, rural contextual constraints, pedagogical adaptations, appropriate *āsana* and *prāṇāyāma* practices, and trainer responsibilities. Trainers described aggression regulation as embedded within routine yoga practice through progressive sequencing of postures, breath regulation, meditation, and relaxation techniques. These insights informed the development of a practitioner-informed framework, along with nine practice recommendations and four operational requirements for safe and context-sensitive yoga delivery in adolescent group settings. The framework highlights the potential role of structured yoga programs as preventive mental health strategies that can be integrated into school and community-based adolescent health initiatives in low-resource environments.

**Conclusion:**

This study proposes a practitioner-informed framework for delivering group-based yoga sessions that support emotional regulation and aggression management among rural adolescents. The findings translate trainer experiences into practice-oriented guidance for implementing structured yoga programs in school and community settings.

## 1 Introduction

Aggression during adolescence represents a significant public mental health concern with implications for long-term behavioral, educational, and social outcomes (1, 2). Preventive strategies that promote emotional self-regulation in adolescents are increasingly recognized as important components of population-level mental health promotion (3, 4). In low-resource settings where mental health services are limited, schools and community spaces can serve as important platforms for delivering preventive programs. Such settings enable preventive approaches to reach large groups of adolescents through routine educational and community activities, making them particularly relevant for public health interventions.

Early manifestations of aggressive behavior are associated with increased risks of substance misuse, academic disengagement, interpersonal violence, and criminal involvement in adulthood (5, 6). The development of aggression is multifactorial, involving biological (7), neurobiological (8), psychological (9), and environmental influences (7). Research on Adverse Childhood Experiences further demonstrates strong associations between early exposure to adversity and later behavioral dysregulation, health risk behaviors, chronic morbidity, and diminished well-being across the lifespan (10–12). These findings identify adolescence as a critical developmental period during which strengthening emotional regulation may help reduce the persistence of behavioral difficulties (13). Developmental psychopathology research further suggests that aggression during this stage may reflect challenges in emotional regulation and executive control under conditions of heightened socio-environmental stress. Reactive forms of aggression have been associated with impaired affect regulation, threat reactivity, and reduced inhibitory control (14).

In rural and resource-constrained settings, adolescents often face compounded vulnerabilities, including socio-economic stressors, exposure to normalized violence, limited educational infrastructure, and restricted access to specialized mental health services (15, 16). Urban–rural disparities in adolescent mental health services in India are substantial, with shortages of trained professionals and limited availability of school-based psychosocial support programs (17). National estimates indicate that India has approximately 0.7 psychiatrists per 100,000 population and that the treatment gap for mental health conditions approaches 90 percent, leaving many adolescents without adequate professional support (18, 19). These structural constraints highlight the need for preventive approaches that can be implemented within accessible community and educational settings. Such conditions further necessitate scalable, non-pharmacological approaches that can be delivered within schools and community settings while remaining sensitive to local cultural practices and available resources (20, 21).

Yoga is a culturally familiar mind–body practice in India that integrates physical postures (*āsanās*), regulated breathing (*prāṇāyāma*), meditation, and relaxation techniques. Empirical research suggests that yoga practices may support parasympathetic activation, stress reduction, and improved emotional regulation (22, 23). Trauma-informed yoga interventions have demonstrated benefits for adolescents exposed to adversity (24), and school-based yoga programs have shown potential in reducing aggression and enhancing psychosocial functioning among adolescents (25, 26). Evidence suggests that yoga may contribute to the development of self-regulation skills that are particularly relevant during adolescence.

Despite growing evidence regarding the psychosocial benefits of yoga, limited attention has been given to how yoga sessions should be structured and delivered within routine adolescent group settings, particularly in rural and resource-constrained contexts. Existing practice frameworks, such as the Common Yoga Protocol (CYP) developed by the Ministry of AYUSH (27), provide standardized yoga practices suitable for community and school settings. Professional organizations including the International Association of Yoga Therapists and Yoga Alliance provide training standards and competency frameworks for instructors (28, 29). However, they provide limited guidance on adapting yoga sessions to adolescent developmental variability, mixed-ability groups, rural infrastructural constraints, and aggression-sensitive contexts. In the absence of such operational guidance, implementation may rely on individual trainer discretion, potentially leading to variability in quality, safety, and participant engagement.

Addressing this gap is important for ensuring that yoga-based interventions are not only theoretically effective but also practically implementable within diverse educational and community settings. In response to this gap, the present study examines the perspectives of yoga trainers (YTs) working with adolescents and develops a practitioner-informed framework for delivering yoga sessions in adolescent group settings. The study also identifies operational requirements for implementing yoga-based emotional regulation programs relevant to rural adolescent mental health contexts.

## 2 Materials and methods

### 2.1 Study design

This study employed a qualitative descriptive design using open-ended online survey responses analyzed through reflexive thematic analysis. The design was selected to capture experiential perspectives of YTs on adolescent instruction and contextual adaptation in rural settings. A qualitative descriptive approach was considered appropriate, as the objective was to generate practice-oriented guidance grounded in participant experience rather than to develop new theory.

The online survey format enabled participation from YTs located across different regions of India while allowing respondents sufficient time to reflect on and articulate their experiential insights in their own words. Online qualitative surveys are increasingly used in health and education research to capture practitioner perspectives when participants are geographically dispersed and when conducting synchronous in-depth interviews is logistically difficult (30, 31). This approach was therefore considered appropriate for the present study, as it facilitated geographically and professionally diverse participation while allowing respondents to provide reflective accounts of their teaching experiences at their convenience.

### 2.2 Participants and sampling

Participants were purposively selected YTs in India who had experience teaching adolescents in school, community, or institutional settings. The inclusion criteria required prior experience conducting yoga sessions for adolescents in structured group settings. Participants were required to be actively engaged in yoga instruction and to have practical experience working with adolescent groups, ensuring familiarity with group-based teaching contexts. The sample reflected practitioners with varied levels of teaching experience, supporting the inclusion of diverse practitioner perspectives. Among the participants, a subset reported direct experience teaching in rural or community-based settings, including government schools, village-level programs, and outreach initiatives. These experiences informed the contextual insights presented in the findings. Although formal psychological training was not an inclusion criterion, many participants reported experiential familiarity with using yoga practices for emotional regulation through their teaching practice, exposure to yoga therapy principles, or institutional training modules that included mental health components.

Participants were identified through professional yoga training networks, institutional contacts, and practitioner communities involved in yoga education and training across India. Participants were recruited from multiple regions across India through these networks, contributing to diversity in contextual experiences. Potential participants were contacted through email and professional messaging platforms and invited to participate voluntarily in the study. Participants were informed about the researcher’s academic background and the purpose of the study before completing the survey.

Participants represented diverse professional backgrounds, including yoga instructors, yoga therapists, educators, meditation instructors, and individuals trained in naturopathy and integrative health systems. Many participants were affiliated with educational institutions, yoga therapy centers, government initiatives, and community-based programs, indicating structured training and professional engagement in yoga instruction. Although formal academic qualifications (e.g., degree-level training) were not uniformly collected, participants’ professional roles and institutional affiliations indicate structured training and engagement in yoga instruction and related disciplines. The diversity of participant backgrounds—including variation in training traditions, institutional affiliations, and teaching contexts—may have contributed to variability in perspectives. While this heterogeneity enhances the ecological validity of the findings, it may also influence differences in instructional approaches and interpretations of adolescent needs.

A total of 36 trainers were invited to participate. Of these, 31 completed the questionnaire and were included in the analysis, while five individuals did not submit responses. The number of invited participants was informed by feasibility and access within professional networks and guided by the aim of achieving thematic sufficiency and capturing diverse practitioner perspectives rather than statistical representation. The final sample comprised 18 female and 13 male trainers, with a mean age of 36.64 years (SD = 9). Trainers reported varied levels of professional experience in yoga instruction (*M* = 7.99 years, SD = 9.35). The relatively wide variation in years of experience reflects the inclusion of both early-career and highly experienced practitioners, contributing to diversity in perspectives captured in the study. Detailed demographic characteristics of participants are presented in Table 1.

**Table 1:** Demographic data of the participants.

Characteristics	Total (*N* = 31)
Gender	Female: 18 (58.06%), Male: 13 (41.93%)
Age (Mean, SD)	Mean = 36.64, Standard Deviation = 9
Yoga genre	*Aṣṭāṅga Yoga* (*n* = 10)
	*Haṭha Yoga* (*n* = 9)
	*Haṭha Yoga, Śivānanda Yoga, Aṣṭāṅga Yoga* (*n* = 1)
	*Haṭha Yoga, Rāja Yoga* (*n* = 1)
	*Aṣṭāṅga Yoga* and *Haṭha Yoga* (*n* = 1) Therapeutic *Haṭha Yoga* (*n* = 1)
	*Amrita Yoga* (*n* = 1)
	Spiritual Yoga practices (*n* = 1)
	*Tantra* (*n* = 1)
	Yoga therapy (*n* = 2)
	All genres (*n* = 1)
	Both theoretical and physical practices (*n* = 1)
	Philosophy, practice, therapy and applications (*n* = 1)
Experience in Yoga training (years)	0–2 (*n* = 6)
	2–10 (*n* = 15)
	10–20 (*n* = 6)
	20–30 (*n* = 3)
	More than 30 (*n* = 1)
	M = 7.99 years
	SD = 9.35 years
Freelance/Affiliate	Freelance (*n* = 7)
	Affiliated (*n* = 24)
	Freelance & affiliated (*n* = 2)
Institutional affiliation of participants	Institutional affiliation of participants (n = 3)
	Universities and higher education institutions (*n* = 2)
	Government-supported programs (*n* = 1)
	Non-profit/spiritual yoga organizations (*n* = 2)
	Private yoga training institutes (*n* = 1)
	Corporate/wellness organizations (*n* = 2)
Number of early adolescents (13–17 years) trained	0–50 (*n* = 13)
	50–500 (*n* = 10)
	500–1,000 (*n* = 4)
	More than 1,000 (*n* = 4)
	M = 505.06 (approx)

### 2.3 Instrument development

Data were collected using a semi-structured questionnaire developed for this study. The instrument consisted of two sections:

1. A demographic profile (age, gender, years of experience, teaching context)
2. Open-ended questions addressing:

- Challenges in teaching adolescents
- Experiences in rural contexts
- Session structure and duration
- Suitable *āsanās* and *prāṇāyāma* practices
- Approaches to emotional regulation and aggression-related behaviors
- Precautions and trainer responsibilities

The questions were informed by a review of relevant literature and refined through consultation with a psychologist and an experienced YT. The instrument was pilot-tested with four trainers not included in the final sample to assess clarity and comprehensibility. Following this, minor revisions were made to improve clarity and flow. These included (i) simplification of question wording to reduce ambiguity, (ii) reordering of items to improve logical sequencing, and (iii) refinement of open-ended questions to encourage more detailed, experience-based responses. The final questionnaire was administered through Google Forms. The questionnaire required approximately 10 min to complete. Responses were collected between May and November 2023.

### 2.4 Data analysis

Qualitative responses were analyzed using reflexive thematic analysis, incorporating both deductive and inductive coding processes, consistent with qualitative descriptive methodology (32–34). The deductive component was informed by the domains embedded in the semi-structured questionnaire, which addressed adolescent developmental characteristics, rural contextual constraints, pedagogical adaptations, practice selection, and trainer responsibilities. These domains provided an initial organizational structure while allowing flexibility for new insights to emerge. While responses were relatively concise, they reflected cumulative teaching experience and provided focused, practice-oriented insights relevant to real-world implementation.

All responses were exported from Google Forms into Microsoft Excel for systematic organization. The first author (male, aged 29, a PhD scholar and yoga practitioner with an interest in adolescent mental health) conducted repeated readings to achieve immersion in the dataset before engaging in line-by-line open coding. Reflexive awareness of the researcher’s background and potential interpretive influence was maintained throughout the analytic process. Codes were generated directly from participants’ written responses rather than imposed *a priori*. Through iterative comparison across cases, related codes were grouped into sub-themes, which were subsequently organized within broader thematic domains. The coding structure linking codes, sub-themes, and overarching themes was developed iteratively during analysis and is reflected in the thematic framework presented in the Results.

Although a consistent set of open-ended questions was used across participants, thematic sufficiency was determined based on conceptual redundancy in the data rather than the uniformity of the questionnaire. This was observed at approximately 22 responses, beyond which no substantially new themes emerged. Responses from all 31 participants were retained and analyzed to capture less frequent but contextually relevant insights and to ensure a comprehensive representation of practitioner perspectives. While recurrence across responses informed theme development, analytic decisions were guided by conceptual relevance and implementation significance rather than frequency alone. Lower-frequency insights were retained when they held practical importance for safe or context-sensitive delivery. Given the use of a structured open-ended questionnaire, iterative modification of questions was not feasible. However, variation in responses emerged from participants’ diverse experiences and contexts, supporting inductive theme development.

Reflexive engagement was maintained throughout the analytic process. Given the first author’s dual background in yoga practice and adolescent mental health, ongoing attention was paid to the potential for interpretive alignment with participants’ perspectives. To address this, coding and interpretation were continually grounded in participants’ descriptions through repeated cross-checking of codes with raw data. The evolving thematic structure was discussed with the second author (female, aged 58, senior researcher with a PhD) to enhance analytic coherence, credibility, and critical reflection. These processes supported the development of themes that remained closely aligned with participants’ accounts while minimizing the influence of prior assumptions and interpretive bias. The final thematic framework informed the development of structured delivery recommendations presented in the Results section. Reporting of the qualitative study followed established qualitative reporting standards, drawing on the Consolidated Criteria for Reporting Qualitative Research (COREQ), where applicable to online qualitative survey designs (35).

Following theme development, a structured interpretive synthesis was conducted to translate themes into practice-oriented recommendations and a conceptual framework. This involved mapping relationships across thematic domains (developmental, contextual, pedagogical, and practice-related) to identify implementation-relevant linkages.

### 2.5 Ethics statement

The study was conducted in accordance with the Declaration of Helsinki and received approval from the Institutional Ethics Committee of Amrita Institute of Medical Sciences, Amrita Vishwa Vidyapeetham (Approval No: IEC-AIMS-2023-ASSD-128) (36). Prior to participation, all YTs were provided with information regarding the purpose of the study, voluntary nature of participation, confidentiality of responses, and the right to withdraw. Informed consent was obtained electronically through the Google Forms platform; participants were required to indicate their consent before accessing the questionnaire. No personally identifying information was collected, and responses were anonymized for analysis.

## 3 Results

Analysis of responses from 31 YTs generated nine major thematic domains reflecting developmental characteristics of adolescence, contextual constraints in rural settings, pedagogical adaptations, selection of practices, and trainer responsibilities. These themes and associated sub-themes are summarized in Table 2. Numerical counts are provided for descriptive context only and do not represent statistical inference. Representative excerpts are included to illustrate recurring perspectives across participants.

**Table 2:** Themes and sub-themes with scoring for each sub theme.

Themes	Sub-themes
Challenges of training adolescents	Attention span and concentration (*n* = 14)
	Motivation and regularity (*n* = 16)
	Communication and instruction (*n* = 5)
	Parental involvement (*n* = 3)
	Individual differences and skill levels (*n* = 9)
Challenges of training rural adolescents	Awareness and understanding of yoga (*n* = 6)
	Infrastructure and resource constraints (*n* = 9)
	Attendance and scheduling (*n* = 3)
	Concentration and engagement (*n* = 10)
	Language and cultural barriers (*n* = 9)
	Psychological and health challenges (*n* = 5)
Practical tips to teach adolescents	Engagement and relevance (*n* = 14)
	Communication and connection (*n* = 9)
	Motivation and ownership (*n* = 3)
	Tailored approach (*n* = 9)
	Empowerment and skill development (*n* = 7)
Yoga as complementary to traditional education system	Holistic development and well-being (*n* = 17)
	Academic benefits (*n* = 6)
	Incorporating yoga philosophy (*n* = 4)
	Self-realization and mind control (*n* = 10)
	Government and school integration (*n* = 6)
Motivate adolescents for sustained practice	Awareness and Propaganda (*n* = 17)
	Interactive and fun approach (*n* = 10)
	Building a supportive community (*n* = 5)
	Customized approach (*n* = 5)
Ideal duration/session	1 h in the morning (*n* = 8)
	45 minutes to 60 minutes (*n* = 6)
	30 minutes in the morning (*n* = 9)
	Morning (*n* = 9)
*Āśana* suitable for adolescents	*Sūrya Namaskāra* (Sun Salutation) (*n* = 9)
	*Vṛkṣāsana* (Tree Pose) (*n* = 10)
	*Bhujaṅgāsana* (Cobra Pose) (*n* = 9)
	*Trikoṇāsana* (Triangle Pose) (*n* = 5)
	*Pādahastāsana* (Forward Bend Pose) (*n* = 6)
Yoga techniques suitable for adolescents in reducing aggression	*Prāṇāyāma* (*n* = 16)
	Meditation (*n* = 8)
	Yogic breathing (*n* = 10)
	*Śavāsana* (*n* = 5)
	*Yoga Nidrā* (*n* = 3)
	*Sūrya Namaskāra* (*n* = 3)
	Yogic counseling (*n* = 1)
YTs’ caution while training adolescents	Knowledge and techniques (*n* = 7)
	Structured and safe approach (*n* = 10)
	Holistic approach (*n* = 7)
	Positive and gentle approach (*n* = 6)
	Clear communication (*n* = 4)
	Age-appropriate training (*n* = 6)
	Flexibility and supervision (*n* = 6)

### 3.1 Developmental challenges in training adolescents

Participants consistently described adolescence as a stage marked by fluctuating attention, variable motivation, and strong peer influence. These characteristics were viewed as directly affecting engagement, consistency in attendance, and adherence to structured practice. Trainers reported that maintaining focus during sessions required deliberate pacing and interactive teaching strategies.

One trainer summarized these concerns:

“*Their shorter attention spans, the need to maintain motivation, effective communication, distractions from technology, accommodating varying skill levels, managing classroom behavior, and ensuring parental involvement.*” (P12M)

Heterogeneity within groups was also emphasized. Differences in flexibility, emotional maturity, discipline, and prior exposure to yoga, required instructors to adapt sequencing and intensity. Trainers noted that adolescents sometimes attempted to overperform in competitive settings, making supervision and gradual progression essential for safety.

### 3.2 Contextual constraints in rural settings

Trainers with rural teaching experience described infrastructural and socio-cultural factors that influenced session delivery. Limited access to appropriate practice spaces, unavailability of mats, and irregular attendance were frequently mentioned. These constraints often required modification of planned sequences or session duration.

As one participant observed:

“*Students are not focused and poor infrastructure like non availability of mats.*” (P1M)

Limited awareness of yoga benefits and unfamiliarity with Sanskrit terminology were also reported. Trainers described the need to simplify language and adapt communication to local contexts. Emotional vulnerabilities, including low self-confidence and stress, were perceived as more visible among few rural adolescents, necessitating patient engagement and supportive communication.

### 3.3 Pedagogical adaptations and engagement strategies

Responses indicated that experiential and dynamic physical movement-oriented sessions as more effective than theory-loaded instructions. Trainers emphasized explaining practical benefits of *āsana* and breathing techniques to enhance relevance and motivation.

One trainer stated:

“*Being able to connect with the students is very important … It is important to understand their lifestyle and adapt the practices to their requirements so that they find it relevant.*” (P2M)

Building rapport outside formal sessions was viewed as beneficial for understanding students’ challenges and preferences. Gradual progression, humor, encouragement, and adaptation to individual capability were repeatedly mentioned as strategies to maintain engagement.

### 3.4 Yoga as complementary to formal education

Trainers frequently described yoga as supportive of concentration, emotional balance, and discipline within school settings. Rather than positioning yoga as separate from academic education, participants viewed it as strengthening students’ capacity to focus and manage stress.

As expressed by one trainer:

“*Yoga philosophy includes physical, mental and spiritual aspects of an individual. It provides a holistic approach to education that goes beyond academics.*” (P7F)

Several participants suggested integrating short daily routines, such as *Sūrya Namaskāra* and brief meditation, into the school curriculum.

### 3.5 Strategies to promote sustained practice

Maintaining long-term engagement was described as something that is dependent on perceived benefit and classroom climate. Trainers emphasized demonstrating tangible outcomes, such as improved calmness or concentration, to encourage continued participation.

One participant explained:

“*By detailing the physical, emotional, therapeutic benefits of doing yoga … and showing its result help them to continue and sustain practice.*” (P12F)

Interactive sessions, peer encouragement, and a non-judgmental atmosphere were considered important for retention. Flexibility in scheduling and adapting communication style to student personality were also highlighted.

### 3.6 Duration and timing of sessions

Morning practice was commonly preferred due to fewer distractions and perceived greater alertness. However, participants emphasized flexibility based on student interest and context.

As one trainer noted:

“*The ideal time duration for a teenager to effectively practice yoga in a single session typically ranges from 30 to 60 minutes*.” (P12M)

Shorter durations were suggested for beginners, with gradual extension as interest developed.

### 3.7 *Āsanās* suitable for adolescents

Frequently recommended postures included *Sūrya Namaskāra, Vṛkṣāsana, Bhujaṅgāsana, Trikoṇāsana, and Pādahastāsana*. Trainers described selecting practices that promote balance, flexibility, and strength while remaining accessible to mixed-ability groups.

One participant listed:

“*Sūrya Namaskāra, Vṛkṣāsana, Bhujaṅgāsana, Setubandhāsana*.” (P5F)

Safe sequencing and avoidance of excessive strain were repeatedly emphasized.

### 3.8 Practices supporting emotional regulation and aggression management

Breath regulation techniques, meditation, and guided relaxation were identified as particularly relevant for emotional calming and impulse regulation. *Prāṇāyāma* practices such as *Nāḍī Śuddhi, Anuloma-Viloma, Ujjāyī*, and left nostril breathing were frequently mentioned.

A participant noted:

“*Prāṇāyāma - Nāḍī Śuddhi, left nostril breathing, sectional breathing, Nādanusandhāna etc.*” (P11M)

Trainers described aggression regulation as embedded within regular sessions rather than delivered as a separate intervention. Gradual sequencing, breath awareness, and reflective practices were viewed as contributing to improved emotional control over time.

### 3.9 Trainer responsibilities and precautions

Participants emphasized the importance of sequential progression, clarity in instructions, and close supervision. Avoiding unhealthy competition and preventing overexertion were considered critical in adolescent groups.

As one trainer stated:

“*The trainer should have the proper knowledge, techniques on the āsana and breathings. And the way they explain should be in a proper and sequential order.*” (P1F)

Positive communication, awareness of developmental sensitivity, and emotional support were also highlighted.

### 3.10 Proposed practitioner-informed recommendations

The practitioner-informed recommendations were derived through systematic mapping of themes to implementation-relevant domains. The themes identified in the analysis informed recommendations for delivering yoga sessions to adolescents in group settings. These recommendations reflect trainers’ experiences related to adolescent developmental characteristics, rural contextual challenges, instructional approaches, and the selection of appropriate yoga practices. The resulting recommendations are presented in Table 3.

**Table 3:** Practice recommendations for delivering yoga sessions to adolescents.

No	Recommendations
1	Establish consistent routines while adapting instruction to varied ability levels in order to maintain attention and encourage regular participation.
2	Adapt delivery to rural contexts by ensuring availability of basic facilities, using locally understandable language, respecting cultural norms, simplifying terminology, and responding sensitively to adolescents’ physical and emotional needs.
3	Build supportive relationships with adolescents, interact beyond formal sessions where feasible, tailor practices to individual capacities, involve local support when possible, and include activities that strengthen confidence and ownership.
4	Integrate yoga into school routines by emphasizing its contribution to holistic development, stress reduction, improved concentration, and readiness to learn, including feasible daily practices such as *Sūrya Namaskāra*.
5	Encourage sustained participation by clearly communicating the purpose and benefits of practices, creating engaging sessions, and fostering inclusive, non-judgmental group environments.
6	Prefer morning sessions where possible and allow flexible duration, beginning around 30 min and gradually extending toward 45–60 min as interest and capacity increase.
7	Select safe and age-appropriate postures such as *Sūrya Namaskāra*, *Vṛkṣāsana*, *Bhujaṅgāsana*, *Pādahastāsana*, and *Trikoṇāsana* within structured sequences.
8	Include breath regulation, relaxation, and meditation practices—including forms of *prāṇāyāma* and guided relaxation (*Yoga Nidrā*, *Śavāsana* etc)—to support emotional balance and reduce aggression-related responses.
9	Progress gradually, prevent injury, and protect emotional well-being by avoiding excessive intensity or unhealthy competition, and by providing clear, supportive, and sequential instruction.

In addition to these recommendations, several operational requirements were identified to support the safe implementation of yoga sessions with adolescents. These include informed consent procedures, trainer preparedness, supervision, environmental safety, and basic practice conditions. The operational requirements are summarized in Table 4.

**Table 4:** Operational requirements supporting safe implementation of adolescent yoga sessions.

No	Operational requirements supporting safe yoga delivery
1	Confirm that YTs possess appropriate qualifications and sufficient experience in accordance with recognized standards. Where suitable, trainers with grounding in yoga philosophy may include age-appropriate reflective elements that enhance meaning and motivation.
2	Understand adolescents’ prior exposure to yoga, expectations, motivations, and belief contexts before beginning instruction in order to tailor sessions appropriately.
3	Seek informed consent from parents or guardians and obtain relevant information regarding physical or psychological conditions prior to participation to support safe practice.
4	Encourage adolescents to wear loose, comfortable clothing that supports ease of movement and relaxed breathing during practice.

## 4 Discussion

### 4.1 Principal findings

This study explored the experiential perspectives of YTs working with adolescents and translated these insights into structured guidance for delivering yoga sessions in rural group settings. Trainers described adolescent aggression not as a discrete therapeutic problem requiring a separate intervention, but as intertwined with attention regulation, emotional control, motivation, and peer dynamics in routine group sessions. Accordingly, the findings suggest integrating breath regulation, progressive sequencing of *āsanās*, relaxation, and reflective practices such as meditation within regular yoga sessions rather than treating aggression management as an isolated component.

Developmental variability among adolescents, infrastructural constraints in rural contexts, and the need for adaptive communication emerged as central factors shaping yoga instruction. These findings informed nine practitioner-informed delivery guidelines and four operational requirements designed to support safe and developmentally appropriate yoga practice in group settings. Together, these findings provide practical guidance for delivering yoga sessions to adolescents in resource-constrained rural settings.

### 4.2 Integration with existing literature

Research examining yoga-based interventions among adolescents has reported improvements in stress reduction, attention, emotional regulation, and psychosocial well-being following structured programs (25, 37–40). Qualitative and mixed-methods studies have also highlighted the feasibility and acceptability of school-based mind–body programs in supporting youth mental health and behavioral functioning (41–43). However, much of the existing literature focuses primarily on evaluating intervention outcomes rather than describing how yoga sessions should be structured and delivered in diverse educational or community settings.

Standardized practice frameworks, such as the CYP developed by the Ministry of AYUSH (27), provide structured sequences of *āsanās*, *prāṇāyāma*, meditation and relaxation techniques for public dissemination. Similarly, professional bodies including the International Association of Yoga Therapists (28) and Yoga Alliance (29) define training standards and competency frameworks for instructors. While these frameworks support standardization of practice and instructor preparation, they offer limited guidance on adapting yoga delivery to adolescent developmental characteristics, mixed-ability groups, or rural implementation constraints. Guidelines developed by institutions under the Ministry of AYUSH, such as the Morarji Desai National Institute of Yoga, highlight age-appropriate, non-competitive, and safety-oriented approaches to teaching yoga among adolescents. These guidelines emphasize adapting practices to developmental needs and group contexts. The practitioner-informed insights from the present study are consistent with these principles, particularly in emphasizing flexibility, safety, and contextual adaptation in group-based yoga sessions (44).

The present study reflects components commonly implemented in group-based instructional settings, such as *āsana*, *prāṇāyāma*, meditation and relaxation practices. Other elements of yoga, including *yama*, *niyama*, and *mudras*, are integral to the broader philosophical and theoretical framework of yoga as described in classical texts and contemporary practice guidelines. However, these elements were less frequently operationalized in routine adolescent group sessions and were therefore not emphasized in the current practitioner-informed framework. This focus is also consistent with commonly used structured yoga programs, such as the CYP developed by the Ministry of AYUSH and adolescent-focused guidelines developed by institutions such as the Morarji Desai National Institute of Yoga, which emphasize accessible, practice-oriented components suitable for group-based delivery (27).

### 4.3 Developmental and theoretical considerations

Participants consistently described adolescence as a stage characterized by fluctuating attention, peer influence, and varying motivation. These observations align with neurodevelopmental research indicating ongoing maturation of executive control processes and heightened socio-emotional sensitivity (45). Trainers emphasized gradual progression, supportive communication, and flexible session structuring as important strategies for maintaining engagement and safety during practice.

Trainers frequently identified *prāṇāyāma*, progressive sequencing of *āsanās*, meditation, and relaxation practices such as *Śavāsana* and *Yoga Nidrā* as supportive of impulse regulation and emotional awareness. These perspectives are consistent with models of self-regulation suggesting that repeated attentional training and breath regulation may strengthen inhibitory control and reduce reactive responding (25, 26, 45, 46).

**Figure 1:**
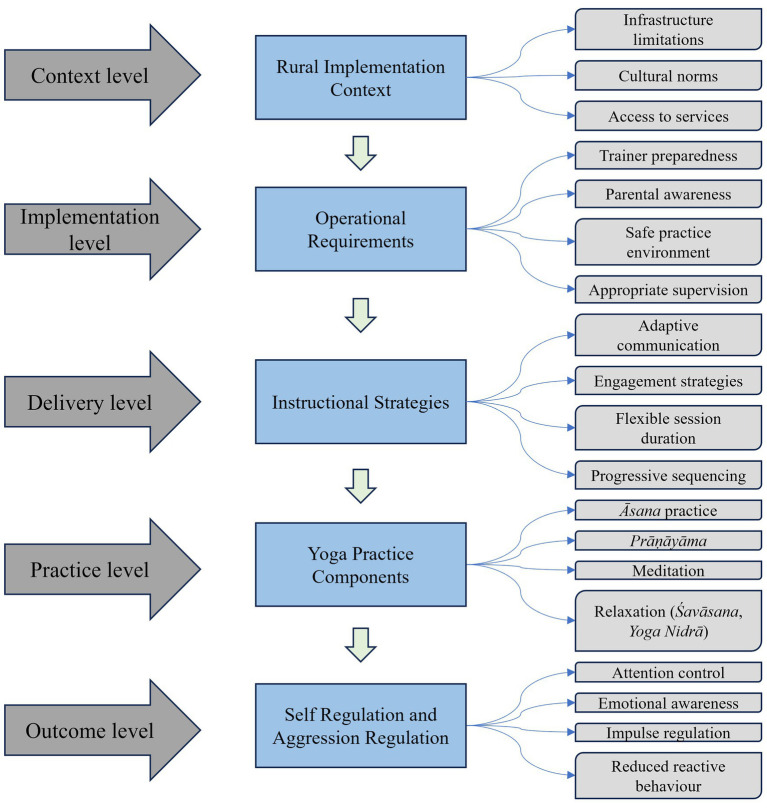
Practitioner-informed framework for yoga delivery supporting emotional regulation among rural adolescents.

The thematic patterns identified in this study indicate that yoga-based emotional regulation in adolescent group settings is shaped by multiple interacting elements. Trainers described contextual influences, implementation conditions, instructional approaches, and specific yoga practices as jointly shaping the delivery and experience of yoga sessions. These relationships are represented in a practitioner-informed framework illustrating how contextual, instructional, and practice-level factors interact to support emotional self-regulation (Figure 1).

Trainers’ accounts suggest that the effectiveness of yoga sessions is contingent upon the conditions under which they are delivered, including contextual factors such as infrastructural constraints, cultural familiarity with yoga, and access to resources, which influence participation and continuity. Operational conditions—including trainer preparedness, appropriate supervision, and safe practice environments—were identified as essential for ensuring responsible and consistent implementation. Instructional strategies, such as adaptive communication, gradual progression, and learner-centered pedagogical approaches, further influence how adolescents engage with and respond to yoga practices. Within this context, practices including *āsana*, *prāṇāyāma*, meditation, and relaxation contribute to the development of attentional control, emotional awareness, and impulse regulation. Collectively, these findings point to a multi-level process in which contextual conditions, operational factors, and instructional strategies interact with yoga practices to shape emotional regulation among adolescents.

The patterns reflected in this framework are broadly consistent with previous research examining yoga-based and mind–body programs for children and adolescents. Studies have reported that practices such as *āsanās*, *prāṇāyāma*, meditation, and relaxation can support improvements in emotional regulation, stress management, attentional control, and overall well-being among young populations (25, 26, 46–49). Research examining school-based health and well-being programs similarly indicates that outcomes are influenced not only by the practices themselves but also by the conditions in which they are delivered. These include trained facilitators, supportive instructional environments, and developmentally appropriate teaching approaches (50, 51). Implementation-focused studies further highlight the role of contextual factors such as institutional support, program organization, and accessibility in shaping participation and sustained engagement in adolescent well-being programs (3, 52). Collectively, this evidence indicates that outcomes in adolescent yoga programs depend not only on the practices themselves but also on the conditions in which they are delivered.

### 4.4 Contextual and rural implementation

The findings of this study should be interpreted within the broader context of structural challenges affecting adolescent mental health care in rural regions. Limited availability of trained professionals, geographic barriers, and uneven service distribution continue to affect access to formal mental health care across many parts of India. These constraints can delay early identification of emotional and behavioral difficulties among adolescents and reduce opportunities for timely psychosocial support. Although national initiatives such as the National Adolescent Health Programme aim to strengthen awareness and coping skills among young people, the reach and continuity of these efforts remain uneven in several rural areas (21).

In many rural communities, health-seeking practices are also influenced by the availability and cultural acceptance of traditional and complementary medical systems recognized under AYUSH, including Ayurveda, Yoga, Unani, Siddha, and Homoeopathy (53). These systems are widely embedded in local cultural traditions and continue to play an important role in community health practices. At the same time, mental health stigma, financial barriers, and shortages of specialized services can restrict access to evidence-based psychosocial care (54–56). Such conditions highlight the importance of preventive approaches that can be implemented within community and educational environments where adolescents already engage in daily activities.

Within this context, the practitioner-informed framework developed in the present study highlights the potential role of structured, group-based Yoga programs as a complementary approach to adolescent well-being. Yoga practices are culturally familiar in many Indian communities and can be delivered in schools, community centers, or youth programs without requiring extensive clinical infrastructure. When implemented with appropriate training, supervision, and safe practice environments, such programs may provide accessible opportunities for strengthening emotional regulation skills among adolescents. While Yoga cannot replace specialized mental health services, it may contribute to preventive public health efforts aimed at supporting adolescent well-being in rural and underserved settings.

While participants were recruited through networks spanning multiple regions across India, detailed regional identifiers were not collected. Given the known variation in socio-economic conditions, infrastructure, and educational contexts across regions, these factors may influence implementation practices. However, the present study aimed to identify cross-cutting patterns in yoga delivery that are relevant across diverse settings.

### 4.5 Study design and analytical approach

The use of an open-ended online survey facilitated participation from geographically dispersed trainers and allowed respondents to provide reflective written accounts based on cumulative teaching experience. While asynchronous data collection limited opportunities for probing, it supported the inclusion of participants from diverse locations and professional contexts (31, 57).

Although recurrence across responses informed theme identification, analytic decisions were not determined by frequency alone. Lower-frequency themes, including parental involvement and individualized adaptation, were retained due to their practical importance for safety and continuity. This approach aligns with qualitative analytic traditions in which conceptual and implementation relevance may outweigh numerical prevalence (34). The brevity of responses reflects the structured nature of practitioner input rather than absence of depth, as participants often provided distilled insights based on accumulated experience.

### 4.6 Strengths, limitations, and future directions

A key strength of this study lies in its practitioner-informed approach and focus on implementation realities rather than solely on intervention outcomes. By synthesizing experiential insights from YTs working with adolescents, the study provides practice-oriented guidance that reflects real-world instructional contexts. This perspective contributes to understanding how yoga-based practices may be delivered in group settings that include developmental variability and resource constraints.

Several limitations should be acknowledged. First, the analysis relied on written responses obtained through an online survey rather than in-depth interviews, which may limit the depth of contextual detail available for interpretation. Second, participants were drawn from a single national context, and therefore the transferability of findings to other cultural or institutional settings should be considered cautiously. Third, the study relied exclusively on practitioner perspectives without incorporating adolescents’ lived experiences. Including adolescent perspectives could provide additional insight into how yoga-based practices are perceived, interpreted, and experienced within group programs. Fourth, the diversity of participant backgrounds may have influenced instructional perspectives and approaches; however, this heterogeneity also enhances the ecological validity of the findings by reflecting real-world variation in yoga instruction. Finally, detailed geographic information of participants was not systematically collected, which limits the ability to interpret regional variations in instructional contexts and practitioner perspectives.

Future research should examine the application of the practitioner-informed framework and associated practice recommendations in school or community settings. Empirical studies evaluating their influence on behavioral, emotional, and psychosocial outcomes among adolescents would further strengthen the evidence base. Incorporating adolescent perspectives in future work may also help refine the framework and improve its relevance for youth populations.

### 4.7 Public health implications

The findings indicate that group-based yoga sessions can incorporate practices that support emotional regulation among adolescents. In rural and resource-constrained regions where access to specialized mental health services is limited, structured yoga programs delivered in schools or community settings may offer a practical preventive approach for supporting adolescent well-being. The practitioner-informed guidelines developed in this study provide practical guidance on session organization, appropriate selection of *āsanās* and *prāṇāyāma* practices, and trainer preparedness and supervision. Such guidance may improve the safety and consistency of yoga programs implemented for adolescent groups.

From a public health perspective, the proposed framework and guidelines may support the integration of yoga-based practices within existing adolescent health and school-based initiatives. In the Indian context, these approaches could be incorporated within school health programs, the School Health and Wellness Programme under Ayushman Bharat, activities implemented through the National AYUSH Mission, and community youth initiatives conducted by educational institutions or local organizations (58–61).

The recommendations are grounded in routine group delivery rather than personalized therapeutic intervention, making them particularly suited to settings where access to mental health professionals remains limited. Incorporating structured yoga sessions into school schedules or community youth programs may provide accessible opportunities for strengthening emotional regulation skills among adolescents. Future implementation research should examine how these practitioner-informed guidelines can be adapted across different educational and community contexts and evaluate their influence on adolescent behavioral and mental health outcomes.

## 5 Conclusion

This study translated the experiential perspectives of yoga trainers into practitioner-informed guidelines for delivering yoga sessions to adolescents in rural group settings. The findings indicate that emotional and behavioral challenges can be addressed within routine yoga practice through appropriate sequencing of *āsanās*, *prāṇāyāma*, relaxation practices, and supportive instructional approaches. The proposed framework and guidelines offer a structured approach for integrating yoga into school and community-based adolescent health initiatives, particularly in resource-constrained settings where access to specialized mental health services is limited. Future research should examine the feasibility, scalability, and behavioral health outcomes associated with implementing these recommendations in diverse educational and community contexts.

## Data Availability

The raw data supporting the conclusions of this article will be made available by the authors, without undue reservation.
