# Supplementary material for: A practitioner-informed framework for yoga-based emotional regulation among rural adolescents
Source: Front Public Health. 2026 May 18;14:1842012. doi: 10.3389/fpubh.2026.1842012 (PMC13223152; doi:10.3389/fpubh.2026.1842012)
Supplement: Supplementary file 1 [file Data_Sheet_1.PDF]

Supplementary Material

1. Semi-structured Interview Schedule

1.1 Instructions for participants

Following are a few questions related only to your experience as a yoga trainer, and your perspectives, observations and suggestions concerning yoga training. Kindly provide your valuable insights. This can help us improve our understanding about evolving a Yoga training module (a part of my PhD research) for adolescents in Indian villages.

Kindly respond to all the questions. We will only be using the information for compilation, without using the identity of the respondent. Hence, kindly do not hesitate to be open about your responses. It will only take 10 minutes to complete the survey. Kindly indicate if you agree or do not agree to participate in the study. (In the Google Form provision was given to the participants to put ✔ to express their consent for participation).

1.2 Demographic Profiler

Name:

Sex:

Age:

Occupation:

Genre of Yoga are you trained in:

Years of experience in Yoga training

Are you a freelance Yoga teacher or are you offering yoga sessions associated with an organization? If Yes, kindly name the organization.

Approximately how many adolescent students (13-17 years) you would have trained in your Yoga career?

1.3 Semi-structured Interview Questions

1) Kindly share with us your challenges, while offering training sessions for adolescent children in India.
2) Kindly share with us your challenges, if you have offered training sessions for adolescent children in rural areas.
3) Kindly share with us the practical tips to teach Yoga to adolescents.
4) Kindly share with us, how Yoga can be considered complementary to the traditional educational system for adolescents in India.
5) As a Yoga trainer, please share with us how you can motivate teenagers in India to join a yoga class and help them continue and sustain practice.

6) Kindly tell us about the ideal time duration per session for a teenager to be effectively doing Yoga.
7) Kindly share with us the list of most important Yoga asanas suitable for adolescents in India.
8) Kindly share with us the list of best Yoga techniques suitable for adolescents in India, to help them control their aggression or anger.
9) Kindly share with us the aspects a Yoga trainer should be careful about while offering training to adolescents.

2. COREQ (Consolidated criteria for Reporting Qualitative research) Checklist

Table 1. COREQ (Consolidated criteria for Reporting Qualitative research) Checklist

**Topic**	**Item No.**	**Guide Questions/Description**	**Reported on Page No.**
**Domain 1: Research team and reflexivity**			
**Personal characteristics**			
Interviewer/facilitator	1	Which author/s conducted the interview or focus group?	NA
Credentials	2	What were the researcher's credentials? E.g. PhD, MD	6, 7
Occupation	3	What was their occupation at the time of the study?	6, 7
Gender	4	Was the researcher male or female?	6, 7
Experience and training	5	What experience or training did the researcher have?	6, 7
**Relationship with participants**			
Relationship established	6	Was a relationship established prior to study commencement?	4
Participant knowledge of the interviewer	7	What did the participants know about the researcher? e.g. personal goals, reasons for doing the research	4
Interviewer characteristics	8	What characteristics were reported about the interviewer/facilitator? e.g. Bias, assumptions, reasons and interests in the research topic	6
**Domain 2: Study design**			
**Theoretical framework**			

Methodological orientation and Theory	9	What methodological orientation was stated to underpin the study? e.g. grounded theory, discourse analysis, ethnography, phenomenology, content analysis	4
**Participant selection**			
Sampling	10	How were participants selected? e.g. purposive, convenience, consecutive, snowball	4
Method of approach	11	How were participants approached? e.g. face-to-face, telephone, mail, email	4
Sample size	12	How many participants were in the study?	4
Non-participation	13	How many people refused to participate or dropped out? Reasons?	4
**Setting**			
Setting of data collection	14	Where was the data collected? e.g. home, clinic, workplace	6
Presence of non participants	15	Was anyone else present besides the participants and researchers?	NA
Description of sample	16	What are the important characteristics of the sample? e.g. demographic data, date	4, 5
**Data collection**			
Interview guide	17	Were questions, prompts, guides provided by the authors? Was it pilot tested?	6
Repeat interviews	18	Were repeat interviews carried out? If yes, how many?	NA
Audio/visual recording	19	Did the research use audio or visual recording to collect the data?	NA
Field notes	20	Were field notes made during and/or after the interview or focus group?	NA
Duration	21	What was the duration of the interviews or focus group?	6
Data saturation	22	Was data saturation discussed?	6
Transcripts returned	23	Were transcripts returned to participants for comment and/or correction?	NA
**Domain 3: Analysis and findings**			
**Data analysis**			
Number of data	24	How many data coders coded the data?	6,7

coders			
Description of the coding tree	25	Did authors provide a description of the coding tree?	6
Derivation of themes	26	Were themes identified in advance or derived from the data?	6
Software	27	What software, if applicable, was used to manage the data?	6
Participant checking	28	Did participants provide feedback on the findings?	NA
Reporting			
Quotations presented	29	Were participant quotations presented to illustrate the themes/findings? Was each quotation identified? e.g. participant number	9-12
Data and findings consistent	30	Was there consistency between the data presented and the findings?	7-13
Clarity of major themes	31	Were major themes clearly presented in the findings?	7-9
Clarity of minor themes	32	Is there a description of diverse cases or discussion of minor themes?	7-14

Developed from: Tong A, Sainsbury P, Craig J. Consolidated criteria for reporting qualitative research (COREQ): a 32-item checklist for interviews and focus groups. *International Journal for Quality in Health Care*. 2007. Volume 19, Number 6: pp. 349 – 357.
